# Orthopedic education: Indian perspective

**DOI:** 10.4103/0019-5413.41848

**Published:** 2008

**Authors:** Sudhir Kumar, SM Tuli

**Affiliations:** University College of Medical Sciences, University of Delhi, Delhi-110 095, India; 1 Vidhyasagar Institute of Mental Health and Neurosciences, Lajpat Nagar, New Delhi, India

Orthopedics as a specialty has grown tremendously in India during the last four decades. High-tech orthopedic delivery has improved the teaching-learning process. However, there is no standardized curriculum for the Orthopedic postgraduate training program. The general guidelines are available with the Medical Council of India (MCI) and the National Board of Examination (NBE).

The postgraduate qualification is imparted by university-affiliated medical institutes. The medical graduates enter the course through a tough competitive entrance examination where orthopedics remains one of the topmost priority courses. The duration of the course is three years. The trainee works as junior resident, writes a thesis/dissertation and appears for a university examination at the end of three years for final assessment in theory, clinical and viva voce. The successful trainee is awarded the degree of Master of Surgery (MS) in Orthopedics.

The National Board of examination runs diplomate of national board in Orthopedics (DNB, Ortho.) in accredited centers in the country. These centers are mostly self-financed/sustained hospitals and not attached with medical colleges. The medical graduate takes up a qualifying examination and an interview to be eligible to undergo three years of training. The trainee maintains a logbook and writes a thesis/dissertation and appears for a theory examination at the end of three years. Once the trainee is eligible to take clinical and viva voce examination in the assigned regional centers; the successful candidates are then awarded DNB in Orthopedics.

## WHAT IS AILING?

The three-year period of in service teaching and training should be utilized to educate an orthopedic trainee about core fundamental knowledge and psychomotor skills to deal with common musculoskeletal clinical problems. What would an orthopedic trainee concentrate to learn, to recall, and to develop psychomotor skills is what he perceives to be assessed and examined by the “prospective examiners”.

The trainees lack a road map in the form of educational objectives which can help them to reach their destinations. The training program does not have structured course content and is mostly trainer-oriented. The trainee is taught what the trainer does the best or the clinical problems specific to that geographic area. The full complement of various subspecialties in orthopedics to provide wholesome postgraduate education is lacking in most of the medical colleges. The system in government hospitals is heavily biased with priorities of service over training component. Hence valuable learning experience is lost as the trainees do not get enough time to discuss the problems with their teacher/mentor. The majority of self-financed/sustained hospitals do not have adequate clinical material to train postgraduates. The trainees are enrolled with those orthopedic surgeons who mainly excel in a certain subspecialty of orthopedics. They may get trained in one aspect of orthopedics and not as competent orthopedists. Most of them don't get enough hands-on training work in these hospitals. There is a great danger that it would create a generation of unsafe orthopedists without a general orthopedic background. There has to be a fundamental knowledge base in basic science and research, core experience in intensive care, trauma, hand surgery, vascular and neurological injuries, musculoskeletal radiology and oncology besides pediatric orthopedics. In most places the delivery of orthopedic education is not organized. The mindset of the teachers/trainers and the examiner must reconcile clearly and loudly that the orthopedic trainee would be examined essentially for fundamentals of core orthopedics. There should be a match between what a student is expected to know and what is asked.

## WHAT IS NEEDED?^1^

1. The current guidelines for three years of postgraduate orthopedic education need to be defined by developing a structured curriculum.
2. The specific guidelines for minimum distribution of education experience in the training program be made mandatory.
3. The conceptual frame work for three years should be laid down so as to define the scope of orthopedic training including the stages, and the milestones that must be achieved by the trainee.
4. A common foundation course for postgraduate is needed to provide an introduction to the principles of education planning, computer literacy, interpersonal skills, medical ethics and legal responsibilities, epidemiology and statistics. The students should also get familiarized with research methodology and literature search techniques.
5. A continuous assessment of clinical competence should be done throughout the program.
6. Psychomotor skill laboratories need to be developed in medical institutions.
7. Technical competence should also be assessed by the ability of formulate a treatment plan for a given clinical problem and should be continuously evaluated to determine the performance in operation theatre and manual skill laboratory.

## HOW CAN WE ACHIEVE IT?

The leadership in this direction has to be taken by the collective efforts of medical institutes in the universities, the Medical Council of India, the National Board of Examinations and the Indian Orthopedic Association. It is suggested that a Orthopedic residency committee (ORC) be formed, which should be empowered to monitor and suggest any changes^2^. They should be able to enforce a uniform residency system of training with a structured national curriculum, ensuring a specific core competency system within the given framework. The assessment tools should truly reflect and linked to this curriculum. It should also be ensured that postgraduate trainees are not used primarily as substitutes for healthcare delivery doctors in the institution.

We also need to be blending the two systems of training in our country. A close relationship has to be developed between the Government Medical College and self-financed hospitals. The accredited centers could be utilized for rotation of trainees in subspecialties. We could utilize the best facilities and human resources to achieve the goal of training a competent orthopedic surgeon.

It is the responsibility of the government to provide adequate infrastructure for medical education in the universities and state medical colleges. The government often underwrites much of the cost of medical education and expects to be well served by the medical graduates. They will have to provide funding to these academic centers for creation of new knowledge and training competent doctors who will improve the healthcare service of the nation.
